# Intrinsic Identification and Mitigation of Multipath for Enhanced GNSS Positioning

**DOI:** 10.3390/s21010188

**Published:** 2020-12-30

**Authors:** Qianxia Li, Linyuan Xia, Ting On Chan, Jingchao Xia, Jijun Geng, Hongyu Zhu, Yuezhen Cai

**Affiliations:** 1 Guangdong Provincial Key Laboratory of Urbanization and Geo-Simulation, School of Geography and Planning, Sun Yat-sen University, Guangzhou 510275, China; liqianx2@mail2.sysu.edu.cn (Q.L.); chantingon@mail.sysu.edu.cn (T.O.C.); gengjj@mail2.sysu.edu.cn (J.G.); zhuhy35@mail2.sysu.edu.cn (H.Z.); caiyzh5@mail2.sysu.edu.cn (Y.C.); 2 School of Civil Engineering, Guangzhou University, Guangzhou 510000, China; jcxia@gzhu.edu.cn

**Keywords:** GNSS, multipath, empirical mode decomposition, frequency spectrum, power spectrum, Hilbert–Huang transform

## Abstract

In global navigation satellite system (GNSS)-based positioning and applications, multipath is by far the most obstinate impact. To overcome paradoxical issues faced by current processing approaches for multipath, this paper employs an intrinsic method to identify and mitigate multipath based on empirical mode decomposition (EMD) and Hilbert–Huang transform (HHT). Frequency spectrum and power spectrum are comprehensively employed to identify and extract multipath from complex data series composed by combined GNSS observations. To systematically inspect the multipath from both code range and carrier phase, typical kinds of combinations of the GNSS observations including the code minus phase (CMP), differential correction (DC), and double differential (DD) carrier phase are selected for the suggested intrinsic approach to recognize and mitigate multipath under typical positioning modes. Compared with other current processing algorithms, the proposed methodology can deal with multipath under normal positioning modes without recourse to the conditions that satellite orbits are accurately repeated and surrounding environments of observing sites remain intact. The method can adaptively extract and eliminate multipath from solely the GNSS observations using intrinsic decomposition mechanism. From theoretical discussions and validating tests, it is found that both code and carrier phase multipath can be identified and distinguished from ionospheric delay and other impacts using the EMD based techniques. The resultant positioning accuracy is therefore improved to an obvious extent after the removal of the multipath. Overall, the proposed method can form an extensive and sound technical frame to enhance localization accuracy under typical GNSS positioning modes and harsh multipath environments.

## 1. Introduction

The global navigation satellite system (GNSS) based technology is rapidly penetrating into various fields, and more and more high performances are preferred. To meet these high-accuracy needs, many efforts have been made to cope with different impact factors including satellite orbit dithering, satellite and receiver clock biases, ionospheric and tropospheric delay, multipath reflections, jamming, and other interferences. These can be classified as influences derived from devices and environments. With technical improvements in precise positioning, most of these impacts can be well modeled and corrected, but not the multipath. Multipath is regarded as a propagation anomaly of line-of-sight signal caused by reflection or diffraction from objects surrounding antenna [1]. It is a side effect relating with scenarios, which may result in time delay for code range, power loss of signal, carrier phase distortion, and phase rate changes relative to line-of-sight signal. For some applications, observation sites can be carefully selected to avoid multipath scenarios. However, it is hard to implement under situations with predefined hardware configurations such as airports, dams, high buildings, and vehicles. Since multipath cannot be eliminated using differential processing or numerical modeling, its impact is obstinate and remains a vital mission for researchers to seek for remedy against positioning loss caused by it in the worse cases [2].

So far, many approaches and techniques were developed to reduce the effect of multipath in GNSS positioning. These can be broadly classified as two types: (1) improvement of antenna and receiver design; and (2) enhancement of data processing skills. For the former, some common techniques such as chock ring antenna, dual-polarized antenna, and the multipath elimination delay lock loop (MEDLL), are used in multipath reduction [3,4,5]. Due to the hardware limitations, most of these techniques do not fit to every positioning system, especially for low cost terminals and boards such as the GNSS embedded in smartphones. Positioning using low cost terminals will thus suffer more accuracy degradation thanks to the multipath. For the latter, many researchers have focused on data processing skills to mitigate the multipath. For example, the signal-to-noise (SNR) based weighting approach is proposed to improve the stochastic model and curb multipath pollution [6,7]. Repeatability based sidereal filtering method is also one of efforts to reduce multipath effect under the condition that antenna and surrounding environment remains intact for some consecutive observing days [8,9,10]. In urban regions, GNSS and digital elevation model (DEM) are jointly used to model environment features to estimate multipath amplitude and delay based on some ray tracing theories [11]. This usually requires support from urban Geographic Information System (GIS) if it is used for practical metropolitical positioning.

The wavelet-based technique is also frequently used in signal and data processing because of its inspective capabilities for time-frequency analysis with flexible scales. Huang et al. [12] applied wavelet to separate multipath from the coordinate domain in dynamic deformation monitoring for high-rise building. It is also employed to explore multipath effects using different forms of observations such as pseudo range, carrier phase, double differential phase, and so on [13,14,15,16]. Pugliano et al. [17] proposed a wavelets-based method to extract and decompose the pseudo range into specular and diffuse one. The wavelet shrinkage was used to reduce high frequency multipath by Souza and Monico [15]. Their work showed different effects were obtained based on six mother wavelets. Since there are different ways to select mother wavelets, it is hard to justify the best one to be used for decomposition and reconfiguration. Similar to the wavelet transform, some filtering approaches—such as the short-time frequency transform (STFT) [18], Vondrak filter [19], adaptive filter [20], and some other methods [21,22,23,24,25]—are also discussed to deal with multipath mitigation. From the abovementioned research items, the filtering results are usually sensitive to selection of control parameters and filter thresholds. Therefore, it is desired for a method which does not require such selections.

In recent decades, the empirical mode decomposition (EMD) has become popular for multipath analysis due to its intrinsic nature and adaptivity for nonstationary signals. Luo et al. [26,27] combined EMD with independent component analysis (ICA) to discover GNSS positioning multipath effect, and then distinguish it from building vibrations in the coordinate domain. In the measurement domain, EMD based schemes were proposed in [28,29] to separate multipath from carrier phase double difference residuals. From summary and analysis for the current developments, there is still lack of sound criterions, to evaluate rationality of the EDM results and proper recognition on each decomposed layer. It is further expected to relate each layer attributes with multipath features that can lead to identification of multipath. These have formed a significant mission for EMD based multipath research. To tackle this dilemma, the Hilbert transform is applied after the EDM to obtain some spectrum consequences regarding signal frequency and energy for the decomposed layers. Multipath is then related with these numerical spectrum consequences and is distinguished from other components such as ionosphere delay residuals, signal bias, GNSS hardware delay, and white noise. These form a motivation for this paper to develop a multipath recognition approach based on data driving pattern with low cost, low complexity, and minimal infrastructure needs [30].

In this submission, multipath for the global position system (GPS) and the BeiDou Navigation Satellite System (BDS), including the Geosynchronous Earth Orbit (GEO), the Inclined Geosynchronous Satellite Orbit (IGSO), and the Medium Earth Orbit (MEO) satellites, are firstly investigated using the combined formula of dual or multiple frequency observations. The investigation serves as basis of understanding and comparisons for subsequent analysis for both single point positioning and differential positioning (DGNSS). To explore code multipath analysis in the case of single frequency, outlines of the EMD and the HHT are given. Their mechanisms to identify and extract multipath are detailed in Section 2. In Section 3, standard point positioning tests in both static and kinematic modes are conducted to verify the efficiency of multipath suppression. This methodology is further extended to facilitate low cost users with only the code range. Our research has exhibited that code range after differential corrections under the DGNSS mode can be effectively purified with elimination of code multipath. The suggested technical frame is then employed to discuss multipath in carrier phase observations. After that, the validating test is given for precise relative positioning combining with short baseline solutions. Conclusions and future planning are finally given in Section 4.

## 2. Materials and Methods

### 2.1. Theory of Empirical Mode Decomposition and Hilbert–Huang Transform

The concept of intrinsic mode function (IMF) was proposed by Norden E. Huang in 1998 aiming at decomposition of any signal into a series of IMFs, which is known as empirical mode decomposition (EMD). After the introduction of Hilbert transform for spectrum analysis, the approach is named as Hilbert–Huang transform (HHT) [31].

The IMF is a stationary function that satisfies two conditions: (1) in the whole data set, the number of extrema and the zero crossings must either equal or differ at the most by one; and (2) at any point, the mean value of upper envelope defined by the local maxima and lower envelop defined by the local minima is zero. The EMD is an adaptive decomposition approach fit for processing of nonlinear and non-stationary signals. Based on time scale features of signal itself, a certain number of IMFs can be obtained that satisfy criteria regarding zero point number, extreme point number, and mean values for upper and lower envelopes for each IMF.

For decomposing a signal *x*(*t*) with EMD method, the following steps are included:

(i) Finding all the local maxima and minima, and connecting the sequential local maxima and minima respectively to obtain the upper envelop *E*1 and lower envelop *E*2 with a cubic interpolation.
(ii) Calculating the mean of upper and lower envelopes *m* = (*E*1 + *E*2)/2.
(iii) Extracting the temporary local oscillation *h* = *x*(*t*) − *m*.
(iv) Repeating (i)–(iii) on the temporary local oscillation *h*, until *h* becomes an IMF denoted as *c*(*t*), which satisfies two conditions for the IMF described before.
(v) Computing the residual, *r*(*t*) = *x*(*t*) − *c*(*t*).
(vi) Repeating (i)–(v) using *r*(*t*) instead of *x*(*t*) to generate the next IMF and residual, until the residual *r*(*t*) becomes monotonic.

Hence, the raw signal *x*(*t*) can be decomposed and reconstructed as

(1) $$x(t)=\sum_{i=1}^{n}c_{i}(t)+r_{n}(t),$$

where *c_i_*(*t*) are IMFs with different local frequencies, obtained in each decomposed layer, which are nonlinear and stationary, and have local features in time domain, i=1,2,⋯,n; and *n* is the number of IMFs. *r_n_*(*t*) is a final residual representing general trend of raw signal.

The IMF is a function that can reflect the intrinsic oscillation of the signal in a single frequency. The frequency of the IMF can be obtained by the Hilbert transform so that a physical explanation of the signal can be provided. ${\tilde{c}}_{i}(t)$, the Hilbert transform for each IMF $c_{i}(t)$ is described as [31]

(2) $${\tilde{c}}_{i}(t)=\frac{1}{\pi }\int_{-\infty }^{\infty }\frac{c_{i}(t)}{t-\tau }d\tau$$

Analytical function $z_{i}(t)$ is constructed as

(3) $$z_{i}(t)=c_{i}(t)+j{\tilde{c}}_{i}(t)=a_{i}(t)e^{j\phi_{i}(t)}$$

where $a_{i}(t)$ and $\phi_{i}(t)$ are the envelop and the phase of $z_{i}(t)$, respectively. From Equations (1) and (3), signal *x*(*t*) can be represented as

(4) $$x(t)=RP\sum_{i=1}^{n}a_{i}(t)e^{j\phi_{i}(t)}=RP\sum_{i=1}^{n}a_{i}(t)e^{j\int \omega_{i}(t)dt},$$

where *RP* is the real part of complex function, and $\omega_{i}(t)$ is the frequency of $z_{i}(t)$. Residual, *r_n_*(*t*), the average trend of the signal, is ignored here. So that Hilbert spectrum and marginal spectrum are obtained as

(5) $$H(\omega ,t)=RP\sum_{i=1}^{n}a_{i}(t)e^{j\int \omega_{i}(t)dt},$$

(6) $$h(\omega )=\int_{0}^{T}H(\omega ,t)dt,$$

where [0, T] is the time span of signal *x*(*t*), and ω is the frequency. Hilbert spectrum, *H*(*ω*, *t*) reflects changes of signal amplitudes within whole time and frequency span. The marginal spectrum *h*(*ω*) is amplitude contribution of individual frequency.

### 2.2. Code Multipath in the Case of Dual Frequency Receivers

In case of dual or multiple frequency observations, code multipath can be exhibited using flexible combinations of code ranges and carrier phases. For example, in dual frequency GNSS receivers, the ionospheric delay in carrier phase can be eliminated with the ionosphere-free combination. The code multipath $MP_{1}$ can be obtained by the combination of observations using (7) and (8) [32,33,34,35,36]

(7) $$MP_{1}=\rho_{1}-\frac{f_{1}^{2}+f_{2}^{2}}{f_{1}^{2}-f_{2}^{2}}\lambda_{1}\varphi_{1}+\frac{2f_{2}^{2}}{f_{1}^{2}-f_{2}^{2}}\lambda_{2}\varphi_{2}+k(N_{1},N_{2}),$$

(8) $$k(N_{1},N_{2})=-\frac{f_{1}^{2}+f_{2}^{2}}{f_{1}^{2}-f_{2}^{2}}\lambda_{1}N_{1}+\frac{2f_{2}^{2}}{f_{1}^{2}-f_{2}^{2}}\lambda_{2}N_{2},$$

where *ρ*_1_ denote pseudo range from receiver to satellite; and *φ*_1_ and *φ*_2_ stand for the carrier phase in L1 and L2 in case of GPS observations, respectively. *N*_1_ and *N*_2_ are the initial ambiguity values of the corresponding carrier phase measurements. (*λ*_1_, *λ*_2_) and (*f*_1_, *f*_2_) are wavelengths and frequencies for (L1, L2), respectively. If the carrier phases have no cycle slips or they have been repaired, *k*(*N*_1_, *N*_2_) with the ambiguity of ionosphere-free combination included will keep constant and can be averaged out in processing.

### 2.3. Code Multipath Identification from Code and Carrier Phase Combination

In case of single frequency, code multipath cannot be exhibited by Formulas (7) and (8). To facilitate multipath analysis and mitigation for single frequency users, code multipath is analyzed based on combination of the code range and carrier phase. The most used form of combination is the difference of the code range and the carrier phase, i.e., code-minus-carrier (CMC). Some issues regarding identification and extraction of the code multipath using an intrinsic data driven model is detailed in this section. The multipath and its relationship with ionosphere delay, carrier phase ambiguity and other biases, are also discussed.

The code-minus-carrier (*CMC*) was formed by subtracting the carrier phase from the code observable as in (9). It is usually used to investigate code multipath for single frequency GNSS receivers. If GPS C/A code range and L1 carrier is used, the *CMC* is formed as

(9) $$CMC=\rho_{1}-\lambda_{1}\varphi_{1}=MP_{1}+2I_{1}+\lambda_{1}N_{1}+\varepsilon_{\rho_{1}},$$

where $\rho_{1}$ denote pseudo range from receiver to satellite; and $\varphi_{1}$ and $\lambda_{1}$ are the carrier phase and wavelength in L1, respectively. $MP_{1}$ is code multipath, and $I_{1}$ is the ionospheric delay in L1. $\varepsilon_{\rho_{1}}$ is the noise of pseudo range. The ambiguity $N_{1}$ will be a constant after cycle slip is detected and repaired. The *CMC* includes code multipath, a twice ionospheric delay and other noises. The code multipath can be identified and extracted by the EMD and the HHT.

### 2.4. Multipath Identification for DGNSS

The positioning accuracy using the DGNSS mode can be improved as the certain types of errors including the satellite orbit error, clock error, and troposphere and ionosphere delay, can be effectively canceled out within a certain range space due to the nature that they are spatially correlated. However, multipath influences on either a base station or a user end, are totally determined by receiver surrounding environments. The multipath impacts on both stations cannot be removed under DGNSS mode. It is therefore essential to investigate code range multipath under this positioning mode. To facilitate the multipath detection under the differential mode, we seek for inspection of code multipath. The code multipath is first obtained from the differential correction (*DC*) data computed at the base station, and the corrected code range on roving user. The *DC* can be expressed as

(10) $$DC=\rho -\rho_{0}=c\delta t^{s}-c\delta t_{r}+I+T+M_{\rho }+\varepsilon_{\rho },$$

where ρ is code range observed on base station; and $\rho_{0}$ stands for geometrical range computed from known coordinates of base station and satellite coordinates acquired from broadcast ephemeris. *c* stands for speed of light. $\delta t^{s}$ and $\delta t_{r}$ are clock errors of the satellite and the receiver, respectively. *M*, *I* and *T* denote multipath, ionosphere and troposphere delay, respectively. $\varepsilon_{\rho }$ is the noise of code range.

The *DC* is used to correct code range for roving user. The corrected code range (*CCR*) will be suffered from both multipath impacts. The *CCR* can be expressed as

(11) $$CCR=\rho_{u}-DC=\rho_{0u}-c\delta t_{r}+c\delta t_{ru}+M_{\rho u}-M_{\rho }+\varepsilon_{\rho u}-\varepsilon_{\rho },$$

where the subscript *u* stands for the user end; $M_{\rho u}$ and $\delta t_{ru}$ indicates code multipath and receiver clock error for the user end. It can be seen that satellite clock and orbital biases, troposphere and ionosphere delays, can be effectively canceled out in *CCR*. The receiver clock biases on both stations are melted together and form a newly mixed clock bias in *CCR* data series. The *CCR* difference among satellites can be further made to remove the mixed receiver clock bias and facilitate EMD decomposition. Similarly, code multipath on both stations are also mixed to form a relative one in *CCR*. Using the EMD and HHT, decomposition of the *CCR* can exhibit and extract relative code multipath. This kind of multipath impact is then removed from *CCR* and so that the purified differential correction can be achieved. Consequently, the influence of code multipath can be eliminated in differential positioning mode.

### 2.5. Multipath Identification for Precise Relative Positioning

The carrier phase is the most precise observation for GNSS based positioning. According to the related research, pollution of multipath may introduce errors to carrier phase as large as one quarter of carrier wavelength [1,12,13]. A heavy multipath scenario thus becomes a main impact source for carrier based precise positioning. Some scholars have suggested wavelet-based techniques and related filtering approaches [17,29,37]. These methods must satisfy some conditions already discussed in Section 1. Therefore, the approach of the EMD and the HHT is employed to inspect and mitigate multipath for carrier phase in relative positioning mode.

To analyze carrier phase multipath in relative positioning, observation model for single carrier observation of the *i*-th satellite is expressed as [34]

(12) $$\lambda \varphi^{i}=\rho_{0}+c\delta t^{si}-c\delta t_{r}-I^{i}+T^{i}+\lambda N^{i}+M^{i}+\varepsilon^{i},$$

where φ denotes carrier phase observation, and $\rho_{0}$ stands for geometrical range between station and the *i*-th satellite. λ is the wavelength and c is the speed of light. $\delta t^{s}$ and $\delta t_{r}$ are clock errors of satellite and receiver. *M*, *I*, and *T* denote multipath, ionosphere and troposphere delay respectively. ε is the noise of carrier phase. *N* is the initial ambiguity value of the corresponding carrier phase measurement.

To remove the existing impacts (e.g., the ionosphere and troposphere delays, receiver and satellite clock biases), double differential observation among stations and visible satellites, is formed as the following linearized expression [34]

(13) $$\lambda \nabla \Delta \varphi_{u,v}^{i,j}=l_{u,v}^{i,j}\delta x+m_{u,v}^{i,j}\delta y+n_{u,v}^{i,j}\delta z+\lambda \nabla \Delta N_{u,v}^{i,j}+\nabla \Delta M_{u,v}^{i,j},$$

where (δx, δy, δz) denotes adjusted baseline vector components; and ($l_{u,v}^{i,j}$, $m_{u,v}^{i,j}$ and $n_{u,v}^{i,j}$) are the coefficients computed from satellite and station coordinates. The ambiguity, $\nabla \Delta N_{u,v}^{i,j}$ is a constant after cycle slip is detected and repaired. For a given baseline solution, carrier phase multipath $\nabla \Delta M_{u,v}^{i,j}$ becomes the relative value between stations and satellites. From Equation (13), it can be seen that this relative carrier phase multipath is directly involved in double differenced carrier phase observations. These observations can be analyzed for discover of multipath using the discussed EMD and HHT methodology.

## 3. Results and Discussion

### 3.1. Investigation of Code Multipath in Case of Dual Frequency Receivers

**Figure 1:**
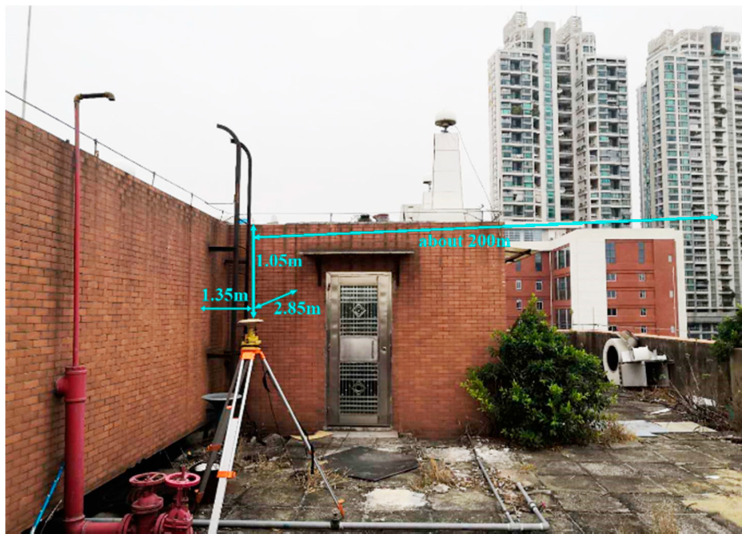
Data collecting site on DOY 310–321, 2018.

To reach an understanding and form the basis of comparisons for subsequent analysis on code multipath—a geodetic receiver, Unicore UR240—was mounted on the roof of Dihuan Building in Sun Yat-sen University (SYSU) for data collection. As shown in Figure 1, there are concrete walls several meters away from the receiver on the northwest and the northeast boundaries, and some buildings are about 60 m high and are roughly 200 m away to the northeast boundary. These obstacles likely cut off satellite signals and cause reflections or diffractions. They are the main sources of multipath in GNSS positioning.

**Figure 2:**
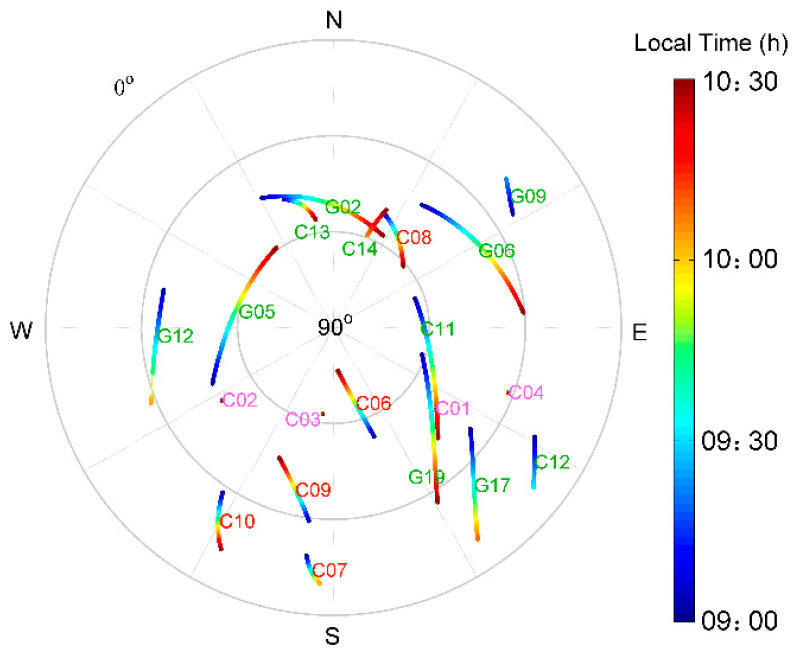
Sky plots on DOY 310 (6 November 2018) from 9:00 a.m. to 10:30 a.m. local on time and observation time spans for each satellite, satellite number in different colors represent type of orbits, for GEO in magenta, IGSO in red, and MEO in green.

**Figure 3:**
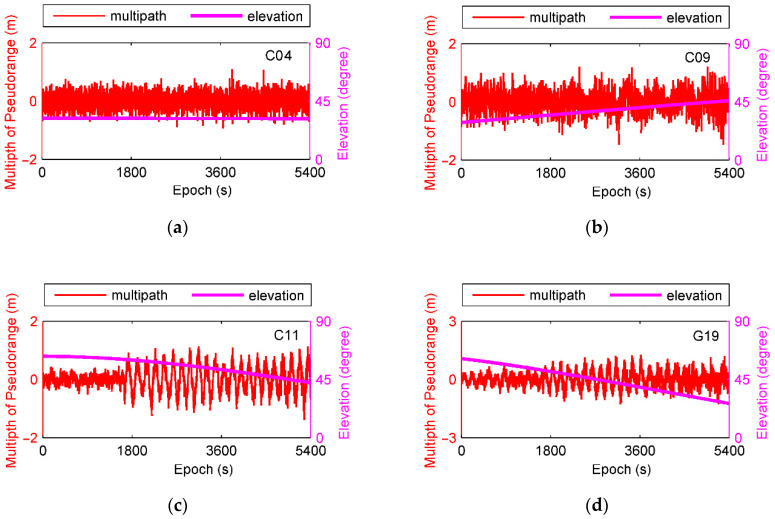
Code multipath and satellite elevations, (**a**) is for C04, (**b**) is for C09, (**c**) is for C11, and (**d**) is for G19.

The GNSS static data were collected from 9:00 a.m. to 10:30 a.m. local time on 6–13 November 2018 (DOY 310-317) at 1Hz sample rate. A sky plot of satellite trajectories on 6 November 2018 is shown in Figure 2. There are four BDS GEO satellites (C01, C02, C03, and C04), and their trajectories are shown as center points as they just swing around the average places. From the northwest to the northeast, satellites with low elevations were invisible due to building blockages. Code multipath was computed using Equation (7) for all BDS and GPS satellites during the observation period on DOY 310–317. Multipath results of BDS GEO C04, IGSO C09, MEO C11, and GPS G19 are given in Figure 3 in which different orbit types of both GPS and BDS are involved with indication of elevations in magenta.

From indicated results, C04 is a GEO satellite moving to and fro gradually, and C09 is an IGSO satellite rising from the southeast. C11 and G19 are MEO satellites sinking quickly. We can see that multipath for GEO satellite is more stable because of its static orbital design. For MEO and IGSO satellites, multipath values become much more noticeable when their elevations are below 45°. It could also be found that multipath for C11 suddenly increases after epoch 1637 even at the elevation of 60° due to the reflected signals from surrounding walls.

**Figure 4:**
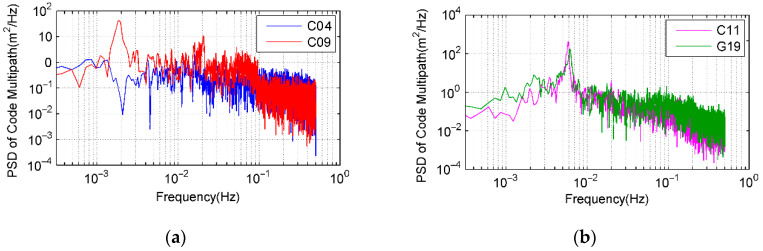
PSD of code multipath (from 9:00 a.m. to 10:30 a.m. local time in DOY 310). (**a**) C04 and C09; (**b**) C11 and G19.

As multipath is caused by interference between reflected signals and line of sight ones, frequency, and power spectrum will become significant attributes for each multipath. Frequency and signal power analysis are used to discover the spectral information for multipath errors. Figure 4 shows the power spectrum of the multipath logarithmic scale for C04, C09, C11, and G19 in DOY 310, which represent BDS GEO, IGSO, MEO, and GPS MEO, respectively. Power spectrum peaks are clearly observed for multipath signals and their changes are even more frequent for IGSO and MEO satellites due to their rapid spatial moving. Power peaks occur approximately between 1 mHz and 20 mHz for both BDS and GPS MEO satellites. The maximum power spectral density (PSD) is around 6 mHz for MEO satellites and 1 mHz for GEO and IGSO ones. After analyzing multipath PSD in other observed days, we can see that MEO satellites of both BDS and GPS have similar power spectrum distributions though they belong to different systems with varied periods. The noises in the BD/GPS data are presented as a combination of white and flicker noises.

**Figure 5:**
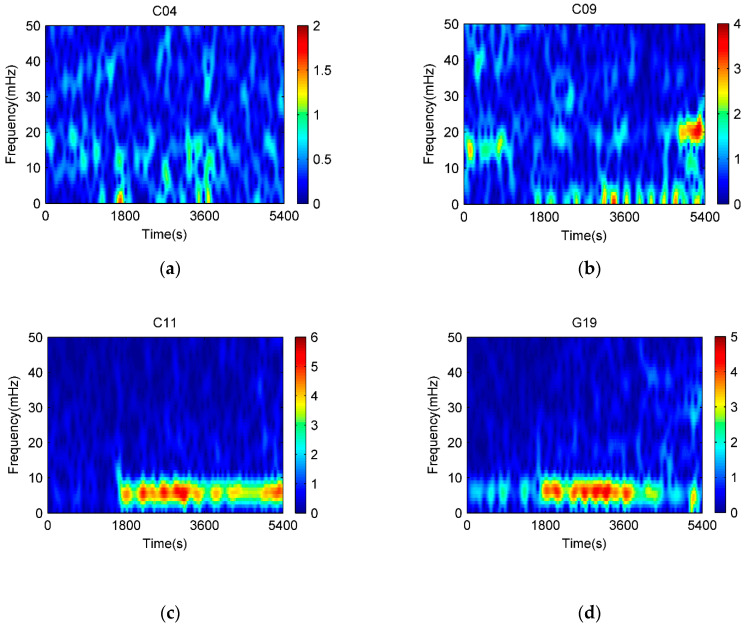
Spectrums of code multipath on DOY 310 (from 9:00 a.m. to 10:30 a.m. local time). (**a**) C04, (**b**) C09, (**c**) C11, and (**d**) G19.

In order to observe temporal changes of multipath central frequencies, frequency spectrum is used and the time-frequency distribution of satellite multipath is shown in Figure 5. It descripts multipath spectrum energy distributions in both time and frequency fields. BDS GEO C04 satellite exhibits an even energy spectrum distribution. This implies that C04 is less affected by multipath since its elevation is relatively high and can overcome multipath to a great extent. In contrast, C11 and G19 have similar power spectrum performances and suffer multipath from 4 mHz to 11 mHz that is determined by scenario of building walls.

### 3.2. Code Multipath Mitigation for Single Point Positioning

**Figure 6:**
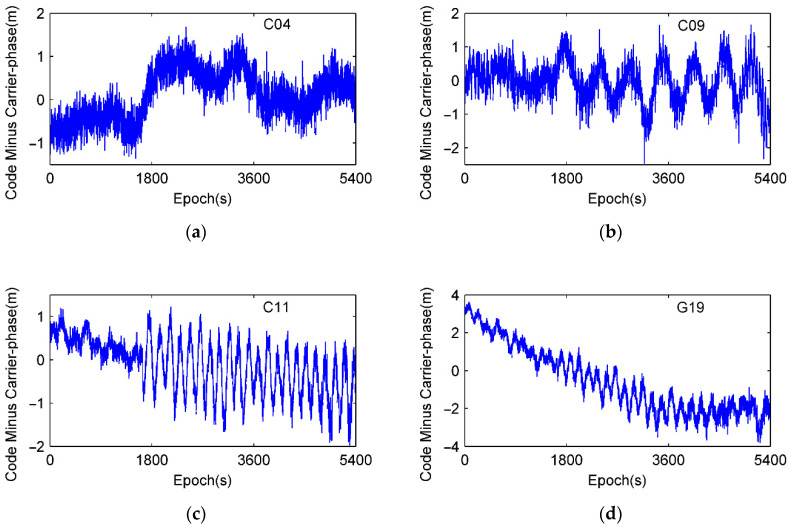
Code minus carrier on DOY 310. (**a**) C04, (**b**) C09, (**c**) C11, and (**d**) G19.

**Figure 7:**
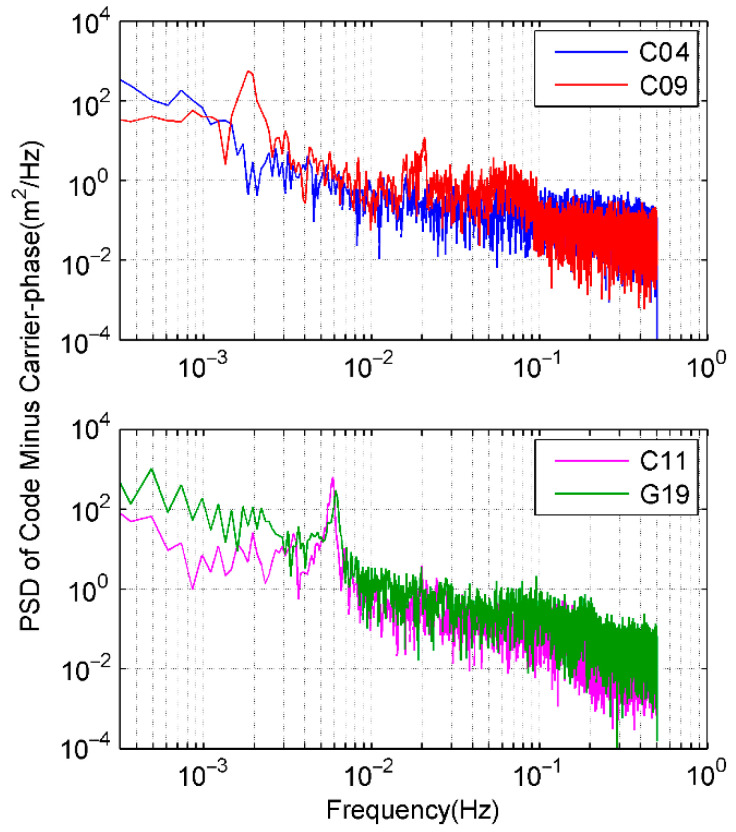
PSD of *CMC* on DOY 310.

For the sake of comparisons with results from dual frequency observations in Section 3.1, the same set of test data is employed and only single frequency observations are used. Figure 6 shows the CMCs of C04, C09, C11, and G19 in the same session of DOY 310. And the relevant power spectrums of the *CMC* logarithmic scale are shown in Figure 7. Compared with Figure 4, four selected satellites have similar PSD peaks between 1 mHz and 20 mHz that reveal the multipath effects. It can also be seen that ionospheric delays mostly have higher PSD values and distribute in the lower frequency parts, usually below the frequency of 1 mHz. Aside from multipath, ionosphere delay, and receiver noise, there are still other impacts including hardware delay and signal bias. To identify and extract code multipath from sophisticated *CMC*, HHT is employed as an optimal approach for multipath inspection from the nonlinear and non-stationary signal *CMC*.

**Figure 8:**
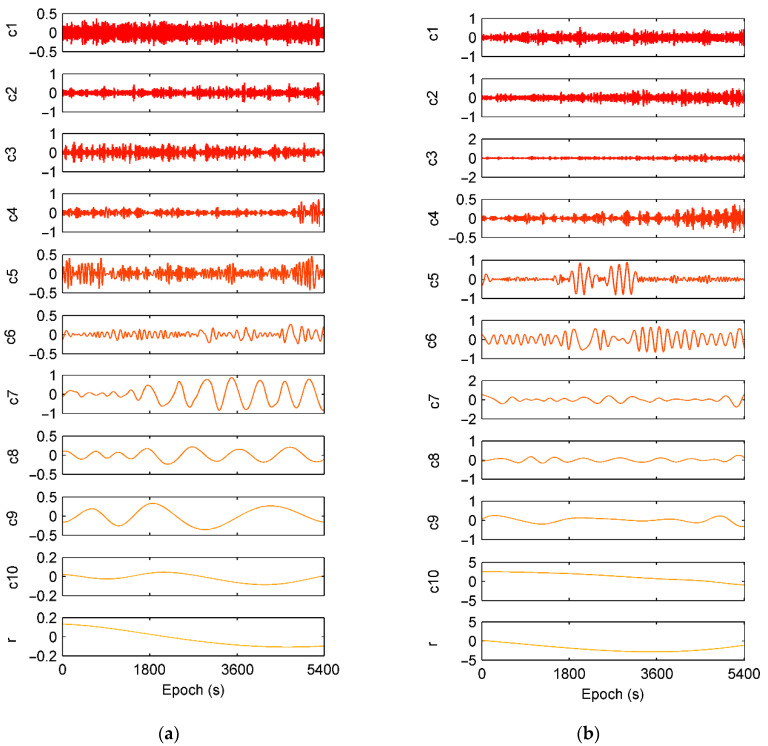
Decomposed layers for *CMC* using EMD approach. (**a**) C09 and (**b**) G19.

**Figure 9:**
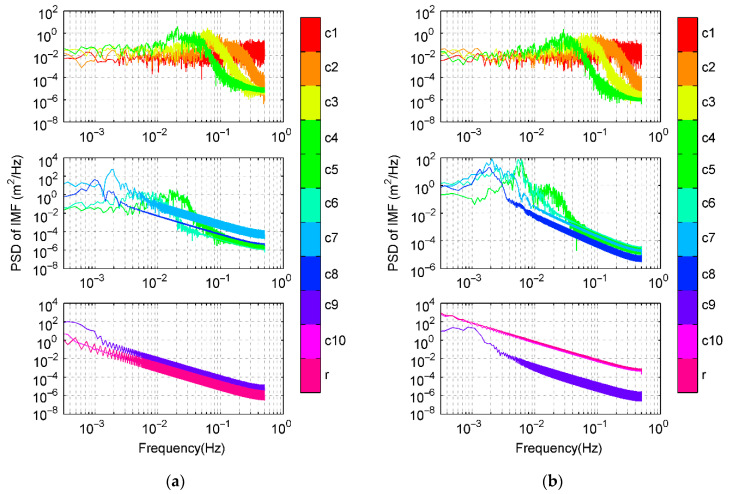
PSD for each decomposed layer from *CMC* using HHT. (**a**) C09 and (**b**) G19.

**Figure 10:**
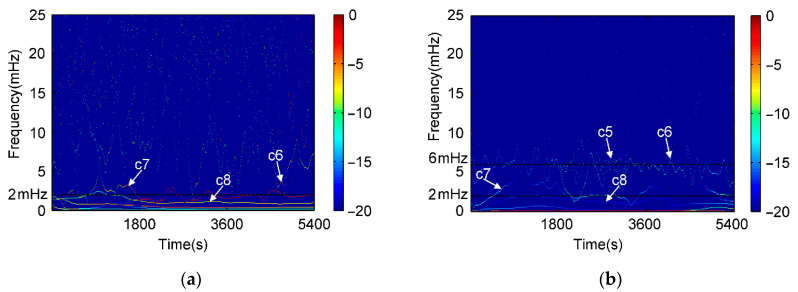
Time-frequency spectrums of IMFs for *CMC*. (**a**) C09 and (**b**) G19.

To seek for understanding and extraction of code multipath from nonlinear *CMC*, EMD is employed to decompose *CMC* data series. The individual IMF components at each decomposition layer of C09 and G19 in the previous experiment of DOY 310 are shown in Figure 8. PSD and HHT time-frequency spectrum of the decomposed results are shown in Figure 9 and Figure 10, respectively. Both C09 and G19 are decomposed as 10 IMFs (c1 to c10) plus a residual r.

**Figure 11:**
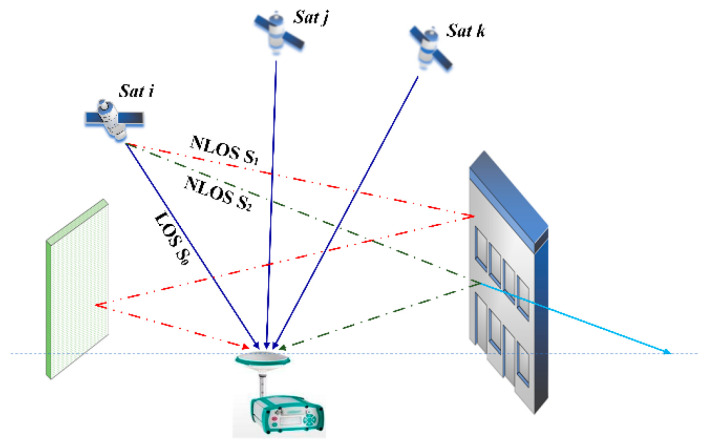
Signal reflection and multipath effects.

From the EMD theory, each decomposed layer is a local frequency component signal with different features. Based on aspects regarding frequency and power spectrum, through comparisons with previous analysis and results, we find that higher and lower frequency components have similar PSD. From c1 to c3 layers, c1 is mainly white noise, c2 and c3 layers are thought to be related with high frequencies of hardware delay and signal bias. In medium frequency spans, different decomposed layers are obtained where code multipath signals mainly distribute which can be verified through PSD shown in Figure 4. This decomposition also implicates that multipath is a mixed component containing reflections and diffractions with varied amplitudes, frequencies, and lasting time determined by multipath scenario. To express corresponding parts in multipath signal, $S_{m}(t)$, which is shown in Figure 11, the summed components can be described as [1,6,13]

(14) $$S_{m}(t)=\sum_{i=1}^{N}S_{i}(t)=\sum_{i=1}^{N}\alpha_{i}A_{i}(t)\cos(\omega t+\theta_{i}),$$

where $S_{i}(t)$ is the *i*-th reflected signal, (i=1,2,⋯,N); and ω is the carrier frequency. $\theta_{i}$ is the phase of the multipath signal due to the *i*-th reflector, which is determined by corresponding reflectivity $\alpha_{i}$. $A_{i}(t)$ denotes the amplitude of the *i*-th multipath contribution.

According to Equation (14) and Figure 8, Figure 9 and Figure 10, it can be deduced that for satellite C09, layers from c6 to c8 are mainly distributing in the frequency of multipath. Layers c5–c8 are code multipath for satellite G19.

**Figure 12:**
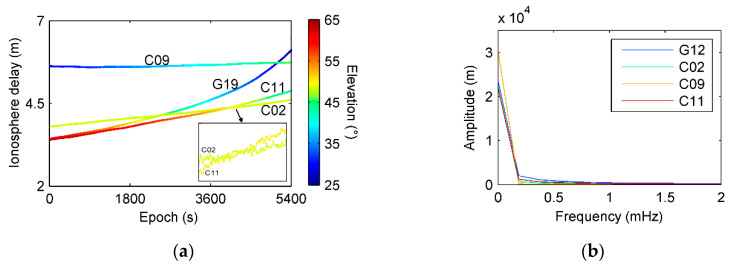
Examples of ionospheric delays and frequency spectrums. (**a**) Ionospheric delays extracted by dual frequency combinations. (**b**) Frequency spectrum of ionospheric delay using FFT.

Lower frequency layers c9 and c10 are mainly concerned with ionosphere delay that has a relatively higher PSD value as shown in Figure 7. Figure 12 demonstrates the behavior of ionospheric delays on the test time, (a) is ionospheric delays of four satellites extracted by dual frequency combinations, and (b) is frequency spectrum of ionospheric delay using FFT. It also verifies that frequency of ionosphere delay is lower than multipath under normal atmospheric conditions. If ionosphere is active, its delay may partly overlap with code multipath and can separate using other filtering approach [26].

**Figure 13:**
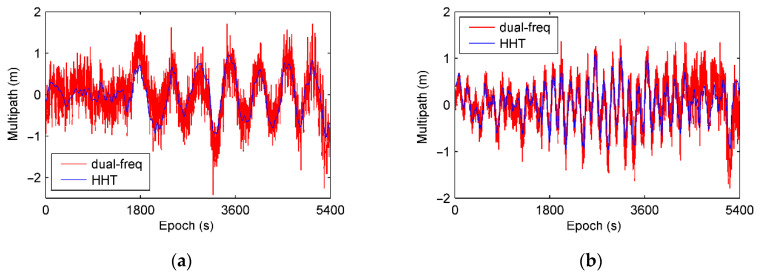
Comparisons of multipath extracted by HHT and computed from dual-frequency combination. (**a**) C09 and (**b**) G19.

Figure 13 shows the code multipath extracted by EMD, and comparisons with results from dual-frequency combinations using Equation (7). From the figure, the multipath extracted by HHT and EMD is identical to that obtained from dual-frequency combination except flutter caused by noise.

**Figure 14:**
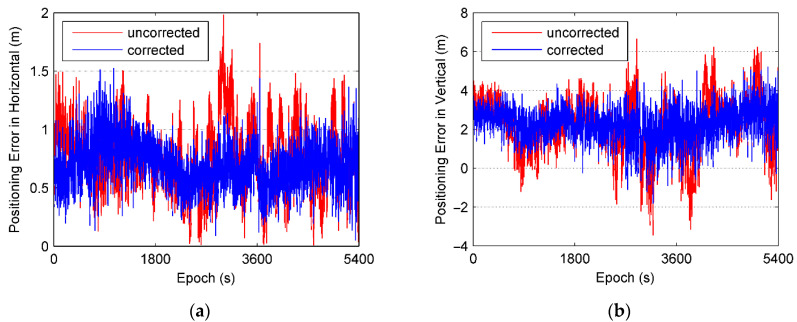
Errors of single point positioning. (**a**) Horizontal error and (**b**) vertical error.

**Figure 15:**
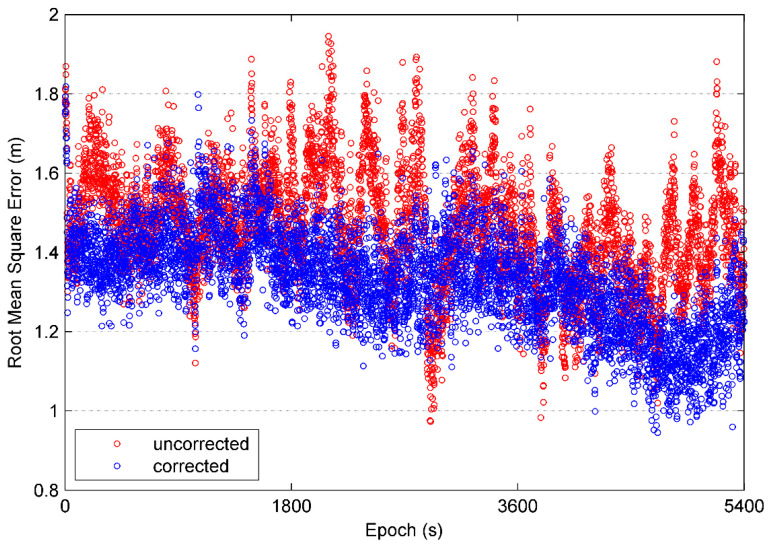
Comparison of root-mean-square error of single point positioning.

**Figure 16:**
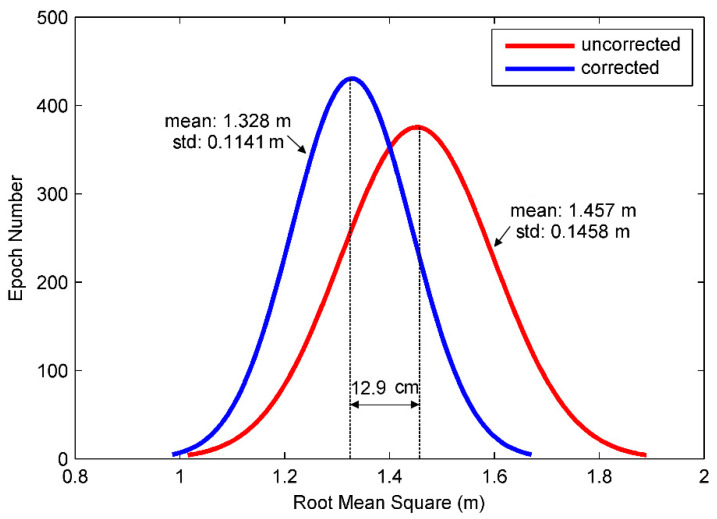
Distribution of root-mean-square error in single point positioning.

To test the efficiency of multipath elimination, BD/GPS positioning using C/A code is carried out. The positioning results are compared with the as reference values obtained from the Trimble Business Center (TBC) baseline solution. The horizontal and vertical errors for the positioning are significantly improved after the multipath correction estimated by the proposed method as shown in Figure 14. The root-mean-squares errors (RMSE) of the positioning is plotted in Figure 15, it can be seen that the RMSE is significantly reduced after the multipath correction is applied. Figure 16 shows a Gaussian distribution of the RMSE, it can be seen that the mean and the standard deviations decrease from 1.457 m to 1.328 m and 0.146 m to 0.114 m, respectively, after the multipath correction is applied. These suggested that our proposed method can improve the accuracy of the positioning suffered from the multipath.

**Figure 17:**
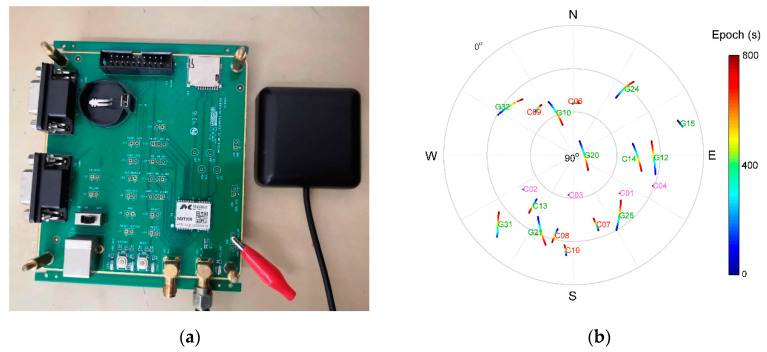
(**a**) Receiver and antenna used for dynamical experiment; (**b**) sky plots of satellites, satellite number in different colors represent type of orbits, for GEO in magenta, IGSO in red, and MEO in green.

**Figure 18:**
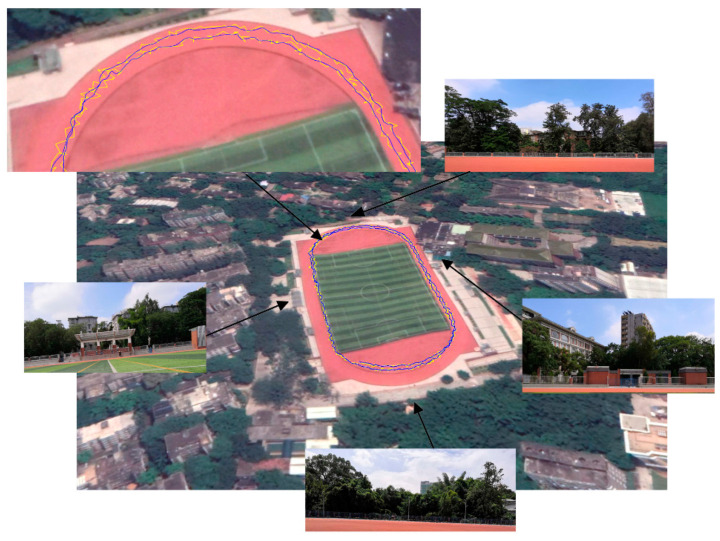
Trajectories of kinematic experiment, result with multipath eliminated is indicated in blue and compared with result influenced by multipath and indicated in yellow.

To further verify multipath mitigation, a kinematic experiment with a low cost BD/GPS navigation receiver and antenna, Mengxin MXT900, in Figure 17a is carried out. For comparison, the precise trajectory is obtained using RTK positioning at the same time together with the navigation receiver that is placed on a handcart and move around the sport ground. There are trees and buildings near the sport ground which may block out satellite signals or reflect signals causing multipath like urban environments. Experiment is carried out on the afternoon of 14 January 2020. Satellites tracked are shown in Figure 17b. Figure 18 gives the surrounding environment and trajectories of the experiment. In Figure 18, blue lines of moving trajectory show the result with multipath mitigated. The other four zoom-in pictures show the surrounding environmental obstacles. Signals reflected by obstacles gave rise to jitter of positioning results. Trajectories become smoother after multipath is identified and removed from code range observations.

**Figure 19:**
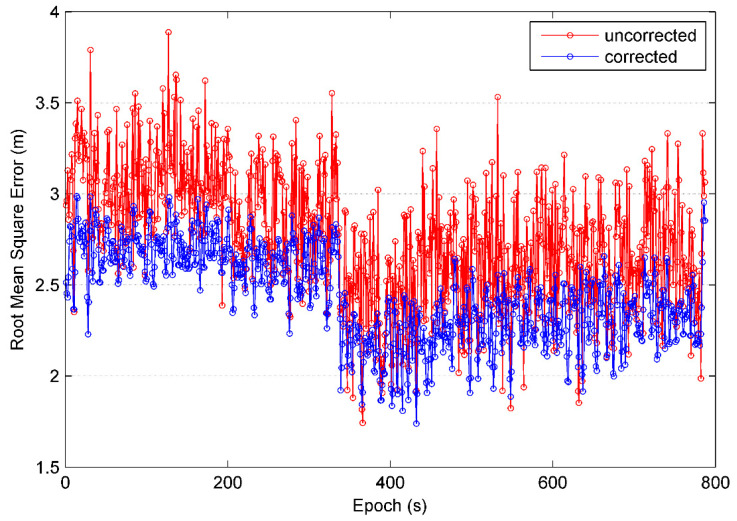
Root mean square error in kinematic single point positioning.

**Figure 20:**
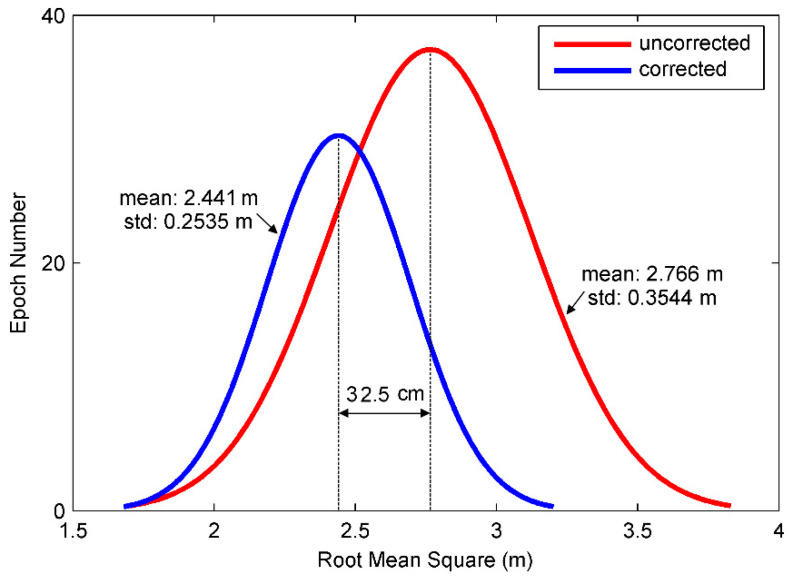
Distribution of root-mean-square error in kinematic single point positioning.

Figure 19 is the rooted mean square error of the kinematic positioning experiment and Figure 20 gives error distribution. The kinematic positioning is given in form of navigation solution of one by one epoch output. In the case that there are some surrounding trees and buildings interfering satellite signals, positioning accuracy of navigation solution using BD and GPS is better than 4 m. After code multipath is identified and mitigated using HHT, accuracy becomes better than 3 m. Average of RMSE is decreased by 12% and its standard deviation is decreased by 28%, from 0.354 m to 0.254 m. From trajectory figures we can see that kinematic trajectories are smoother and more accurate after identification and mitigation of multipath.

### 3.3. Code Multipath Mitigation and Enhanced DGNSS Positioning

**Figure 21:**
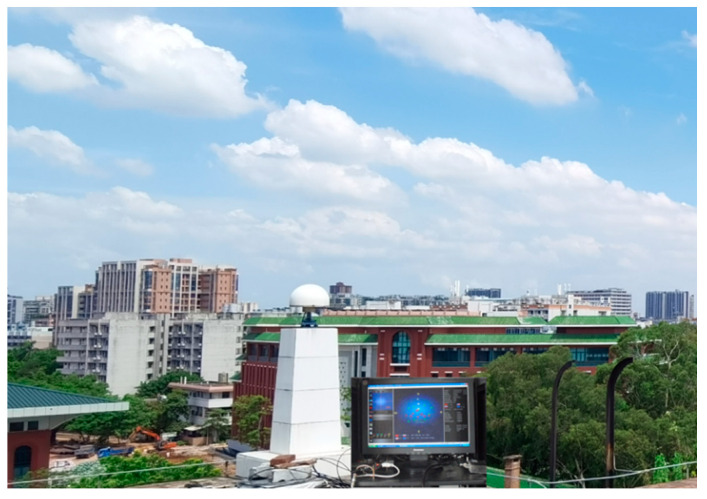
Base station for DGNSS experiment on SYSU campus.

In DGNSS experiment, test data is collected on base station by Unicore UR240 that mounting on top floor of Dihuan Building in Sun Yat-Sen University in Figure 21. Navigation receiver terminal, Mengxin MXT900, in Figure 17a is used as rover user in this test.

**Figure 22:**
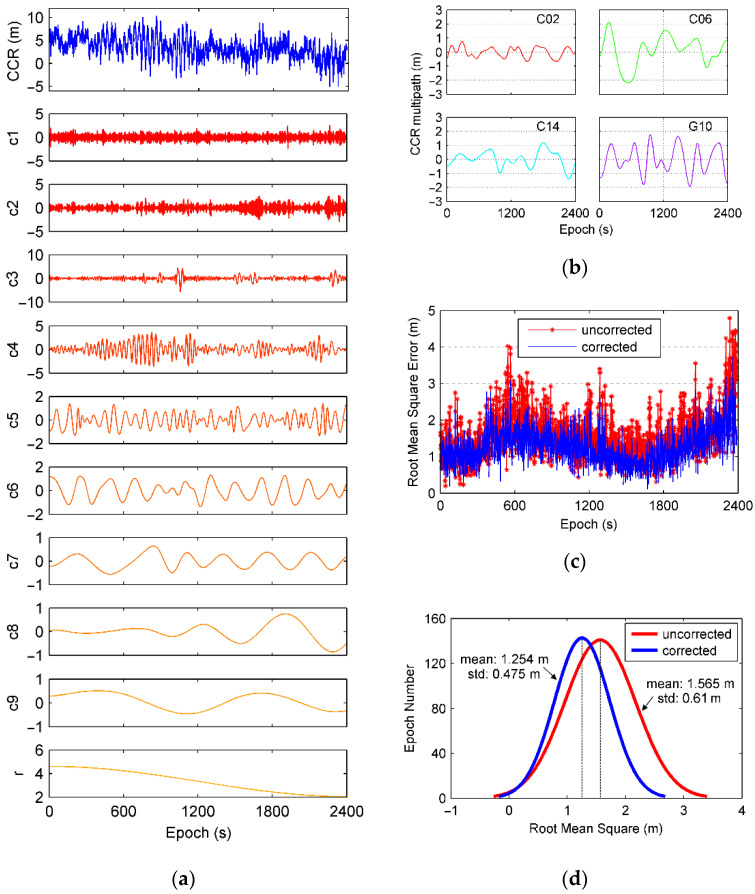
(**a**) Decomposed layers for *CCR* of satellite C14 using EMD approach, (**b**) is multipath of *CCR* extracted, (**c**) is the root mean square error in DGNSS experiment, and (**d**) is the distribution of root mean square for positioning result.

*CCR* (corrected code range) for BDS C14 is plotted as one example in Figure 22a and it is decomposed into nine IMF layers by EMD. Relative code multipath for C02, C06, C14, and G10 are given by Figure 22b. The RMSE of the positioning is plotted in Figure 22c, it can be seen that the RMSE is decreased after the multipath is corrected by the proposed method. Figure 22d shows a Gaussian distribution of the RMSE, it can be seen that the mean and the standard deviations decrease from 1.565 m to 1.254 m and 0.61 m to 0.475 m, respectively after the multipath correction is applied. These suggested that relative code multipath can be identified based on the mechanism discussed previously and be removed from *CCR* to achieve an improved differential positioning result.

### 3.4. Mitigation of Carrier Phase Multipath and Precise Relative Positioning

**Figure 23:**
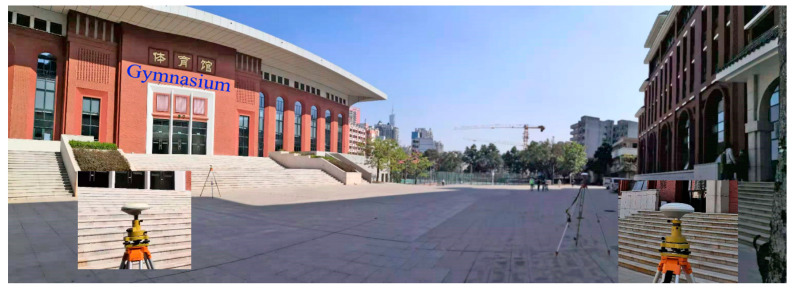
Data collection in SYSU campus for precise relative positioning experiment.

Using the same mechanism for code multipath analysis, the double differenced carrier phase is used as a combination form for phase multipath identification and mitigation. Test data that contain both BDS and GPS observations was collected by Unicore UR240 on Sun Yat-Sen University campus as Figure 23.

**Figure 24:**
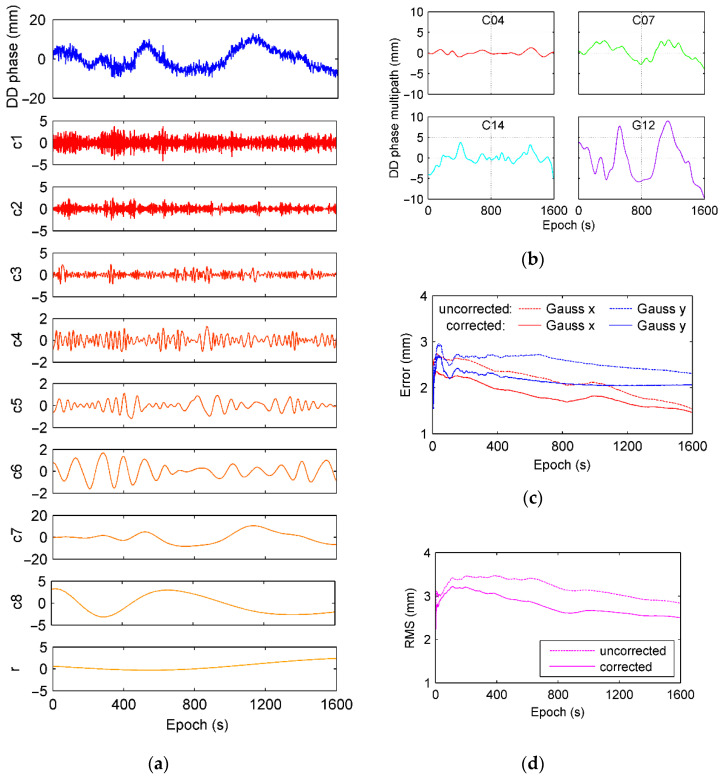
(**a**) Decomposed layers for double differential carrier phase of satellite G12 using EMD approach, (**b**) is multipath of double differential carrier phase extracted by HHT, (**c**) is the root-mean-square error of precise relative positioning, and (**d**) is the distribution of the root mean square.

For short baseline, ionosphere and troposphere delays can be effectively canceled since they are closely and spatially correlated. Carrier noise will be exhibited as high frequency layer of c1, imperfect model error and bias arisen from math/physical correlation under high sample rates are decomposed into lower frequency layers as c7 or c8 in Figure 24a. Double differential carrier phase of G12 in experiment is decomposed into nine layers, including eight IMFs and a residual, showing in Figure 24a. Multipath of double differential carrier phase can be extracted through frequency identified by HHT. The corresponding results from the satellite C04, C07, C14, and G12 are shown in Figure 24b. Similar to code multipath, BDS GEO satellite C04 has smaller multipath in carrier phase because of its orbital characteristics and higher elevation. Multipath effect of GPS G12 is more serious than other three satellites since the satellite is on a lower elevation so that its signal is reflected heavily by buildings around receivers. Figure 24c,d demonstrate errors of baseline solution using BD and GPS. It can achieve horizontal accuracy of 3 mm. With carrier phase multipath identified and corrected by HHT, baseline solution became more accurate.

When the baseline becomes longer, ionosphere and troposphere delays will become less correlated and their impacts cannot be effectively canceled. They are expected to exhibit at certain layers in EMD decomposed outputs. This will be discussed in later research.

## 4. Conclusions

From theoretical aspects, we have combined HHT and power spectrum tools with EMD to form an extensive data driven model. Through analysis and application of the EMD based approach for multipath identification and mitigation, the flexible and intrinsic capability of EMD is exhibited for multiple layer decomposition of nonstationary and nonlinear signals. It is therefore a suitable tool for multipath recognition from complex data series of combined GNSS observations. Based on tests and experiments under typical GNSS positioning modes including single point positioning, code range differential positioning and carrier phase based relative positioning, both code and carrier phase multipath can be extracted and mitigated to an obvious extent to achieve enhanced and accurate results, which has verified the efficiency of our approach. In the future, it is worthwhile to test related issues under longer baselines and precise point positioning mode.

## Data Availability

Data sharing is not applicable to this article.
